# Human *SRD5A1* as a case of gene expression indel-resistance in triple-coding region

**DOI:** 10.1186/s13059-026-04106-x

**Published:** 2026-05-18

**Authors:** Martina M. Yordanova, Jack A. S. Tierney, Kyle A. Meiklejohn, Conor Slattery, Michał I. Świrski, Mirriam Baranova-Gurvich, Manon Engels, Oscar Ting, Ananth Prakash, Håkon Tjeldnes, Jonathan M. Mudge, Gary Loughran, Juan Antonio Vizcaíno, Dmitry E. Andreev, Pavel V. Baranov

**Affiliations:** 1 School of Biochemistry and Cell Biology, University College Cork, Cork, Ireland; 2 European Molecular Biology Laboratory, European Bioinformatics Institute, Wellcome Genome Campus, Hinxton, Cambridge, CB10 1SD UK; 3 Institute of Genetics and Biotechnology, Faculty of Biology, University of Warsaw, Warsaw, Poland; 4 Shemyakin-Ovchinnikov Institute of Bioorganic Chemistry, RAS, Moscow, 117997 Russia; 5 Belozersky Institute of Physico-Chemical Biology, Lomonosov Moscow State University, Moscow, 119992 Russia

**Keywords:** Overlapping genes, Translation initiation, Translon, SRD5A1, uORF, Ribosome decision graphs, Translation control, Protein synthesis, Gene annotation

## Abstract

**Background:**

Nucleotide sequence can be translated in three reading frames producing distinct protein products. Many examples of RNA translation in two reading frames (dual coding) have been identified so far.

**Results:**

We report translation of mRNA transcripts derived from SRD5A1 locus in all three reading frames that result in the synthesis of long polypeptides. This occurs due to initiation at three nearby AUG codons occurring in all three reading frames. Only one of the three proteoforms contains the conserved catalytical domain of SRD5A1 produced either from the second or the third AUG codon depending on the transcript. Paradoxically, ribosome profiling data and expression reporters indicate that the most efficient translation would produce catalytically inactive polypeptide. While phylogenetic analysis suggests that the long triple decoding region is specific to primates, occurrence of nearby AUGs in all three reading frames is ancestral to placental mammals. This suggests that their evolutionary significance belongs to regulation of translation rather than biological role of their products. By analysing multiple publicly available ribosome profiling data and with gene expression assays carried out in different cellular environments, we show that relative expression of these proteoforms is mutually dependent and varies across environments supporting this conjecture. We show that a remarkable feature of triple decoding is its resistance to frameshift causing variants with apparent implications to clinical interpretation of genomic sequence variants.

**Conclusions:**

We argue for the importance of identification, characterisation and annotation of productive RNA translation irrespective of the presumed biological roles of its products.

**Supplementary Information:**

The online version contains supplementary material available at 10.1186/s13059-026-04106-x.

## Background

The redundancy of the genetic code allows for more than one protein sequence to evolve within the same stretch of nucleotides. This property allows to maximize protein coding information content in the confined genomic space of small genomes e.g., those of viruses and bacteria [1–3]. Very short overlaps between coding regions in bacterial polycistronic mRNAs are also common due to coupling of termination and initiation [4]. Because of its bacterial origin [5], mitochondrial genomes (including human) have retained such short coding overlaps. However, the overlap of long coding regions translated in more than one reading frame might appear as an unnecessary complication in larger genomes as its necessity is not imposed by genomic size limitations [6]. Nevertheless, dual coding in the human genome is far from an exception [6–9]. Most examples of overlapping coding regions occur in alternatively spliced transcript isoforms where the same exon is decoded in two different reading frames [7]. For example, alternative first exon utilisation in the INK4a/ARF locus results in the translation of two overlapping frames in the second exon and enables the encoding of two distinct proteins, both with tumour suppressor activity [10].

Alternative translation mechanisms account for a small but growing number of reported dual coding cases. Alternative start site selection in a long transcript isoform from the GNAS locus was shown to result in the synthesis of two functionally related proteins, XLαs and ALEX, from overlapping reading frames in rat [11]. Initiation at a CUG codon supports translation of an overlapping alternative reading frame to generate POLGARF from *POLG* mRNA [12].

Given that genomic space is not a constraint in eukaryotes with large genomes, what are the benefits of encoding proteins in alternative frames of the same RNA molecule? Encoding functionally related proteins in the same locus can enable coordinated expression and response to certain conditions [10]. Also, it could be easier for a new protein to evolve on an mRNA that is already highly expressed than elsewhere in the genome [13].

Overlapping protein coding regions apply specific constraints on sequence evolution and previous studies have assessed certain sequence properties for the identification of dual coding regions [13]. This phenomenon has been suggested to have an evolutionary advantage under conditions of a high mutation rate through superposition of critical points, reducing their amount in the genome [14]. Dual coding regions were also proposed to have a role in the appearance of novel intrinsically disordered regions with new functions [15].

The past decade has seen advances in technologies such as the ribosome profiling that allowed us to obtain an unprecedented high-resolution map of translated regions in the transcriptome [16]. As a result, we can now observe the expression of overlapping regions that were not predicted by sequence analysis alone such as those evolved most recently e.g., specific to humans.

Using publicly available aggregated ribosome profiling data [17–19] we discovered that the same sequence in *SRD5A1* mRNA is translated into three long polypeptides from all three reading frames. This occurs due to initiation at three nearby AUG codons in three different reading frames. By placing gene expression reporters under the control of each individual AUG codon we were able to confirm and characterize this, hitherto unique, example of triple decoding in humans. Strikingly, it appears that the most efficient translation initiation site is an AUG codon not annotated as start, yet our analysis suggests that the corresponding product is unlikely to be functional.

Using naturally occurring genomic variants, we also show that a remarkable feature of triple decoding is its resistance to frameshift mutations. Although the presence of an alternative N-terminus may impair protein activity, a functional protein may still be produced from one of the alternative start codons, especially if the mutation occurs near the beginning of the CDS where changes to the N-terminal sequence would be minimal. This property is also relevant to many other human mRNAs with overlapping translons, where depending on their relative reading phase orientation, either an insertion or a deletion would not result in abolition of protein expression and may even increase it. This is an important phenomenon that needs to be considered during interpretation of genomic sequence variants.

## Results

### SRD5A1 locus and its annotation

**Fig. 1:**
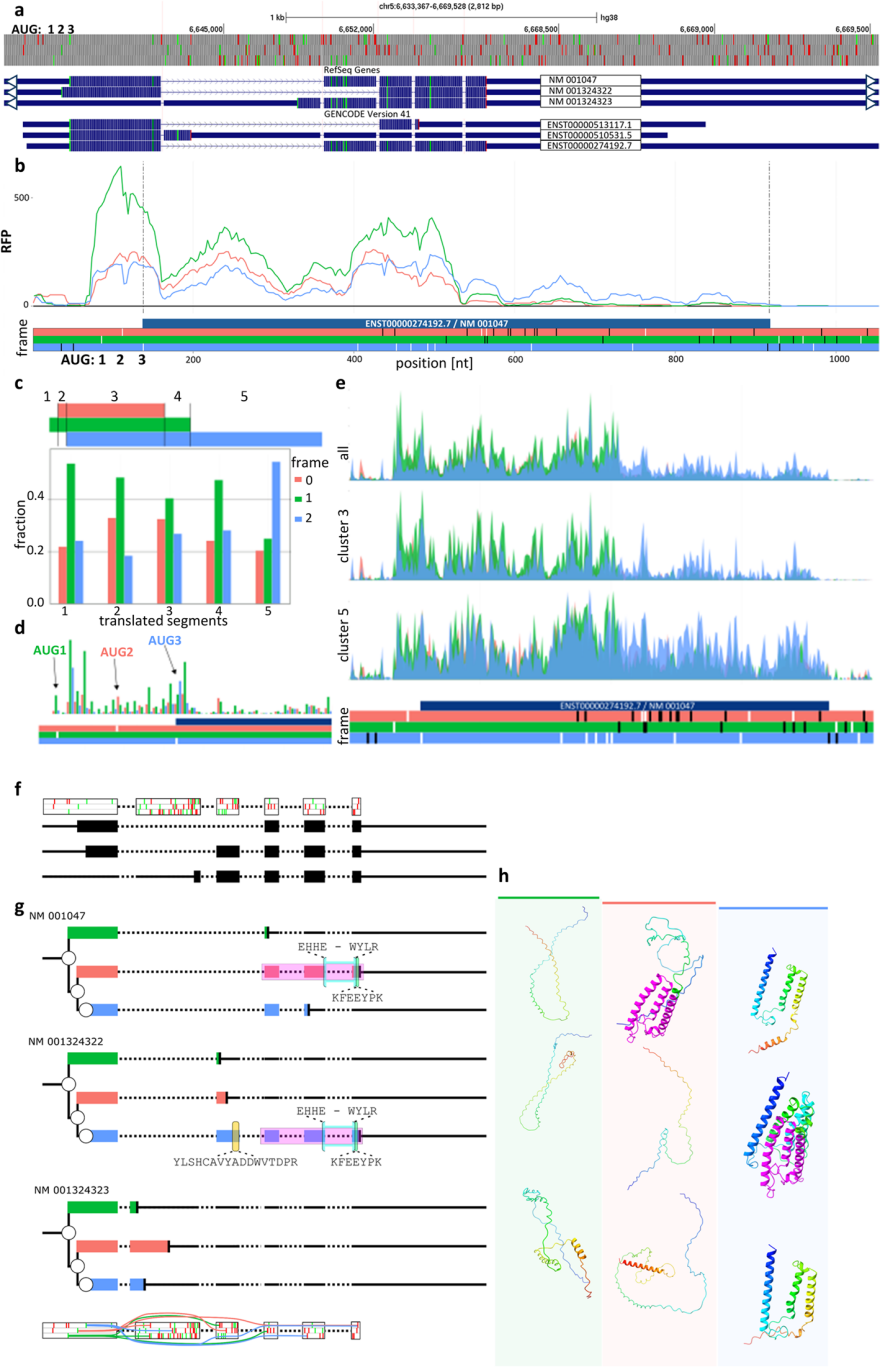
Aggregated Ribo-seq data track provides evidence for translation in three reading frames. **a** Top: open reading frame (ORF) plot with AUG codons in green and stop codons in red. RefSeq and GENCODE *SRD5A1* transcript annotations. Coding and non-coding exon regions are indicated with blue panels of different thickness, introns with thin lines. **b** Ribosome footprint density from aggregated data tracks in RiboCrypt for Ensembl transcript ENST00000274192.7 corresponding to Refseq transcript NM_001047. The line colouring matches the best supported reading frame in the ORF plot below; start codons shown as white bars, stop codons are black. **c** Analysis of Ribo-seq reads density and framing bias in the regions with different overlaps between translons (1–5). The regions with different overlaps are shown at the top. Read densities for each region are shown below separated based on triplet periodicity framing bias. The colours of the columns correspond to the supported reading frames matching the colours of translons at the top. **d** Sub-codon footprint density where in-frame peaks for all 3 AUG codons indicate active translation initiation. Density peaks are coloured to match the best supported reading frame below. **e** Sub-codon coverage plots for two distinct clusters and all samples combined showing differential translation of translons between clusters. **f** Three RefSeq *SRD5A1* transcript isoforms all sharing the first exon but with different AUG codon annotated as a start site. Above is a schematic of the ORF organisation for each coding exon with AUG codons in green and stop codons in red. **g** RDG representations for each of the three transcripts with putative translons indicated. Regions with highlights and dotted annotations show the locations of high-quality matching peptides identified from mass spectrometry data. **h** AlphaFold2 structures for each of the predicted proteoforms. Regions of the translons highlighted in purple indicate encoded SRD5A1 enzymatic activity. Rainbow colouring shows N-terminus (blue) to C-terminus (red) orientation

Steroid 5-alpha-reductase 1 encoded by *SRD5A1* is involved in the regulation of androgen metabolism and is of great interest in the fields of endocrinology and oncology. SRD5A1 converts testosterone into the more potent androgen dihydrotestosterone (DHT). Increased levels and activity of SRD5A1 in prostate cancer result in high DHT levels and castration-resistant prostate cancer [20]. SRD5A1 activity is also elevated in other sex hormone related cancers [21–23]. The *SRD5A1* locus has 6 exons whose alternative splicing gives rise to several transcript isoforms annotated by RefSeq [24] and GENCODE [25] (Fig. 1a). Exon 1 is shared by all annotated transcript isoforms and in it there are 4 AUGs (here and further for simplicity and uniformity we use AUG irrespective whether we refer to sequences of RNA or DNA due to their significance at RNA level). Synthesis of the major annotated SRD5A1 proteoform of 259 AA residues derives from translation initiation at AUG3 in transcript NM_001047/ENST00000274192.7. An alternative proteoform of 212 AA residues with the same C-terminus, is predicted to originate from translation initiation at AUG2 in transcript NM_001324322 lacking exon 3. In transcript NM_001324323, the annotated initiation codon is in exon 2 where it is found downstream to 20 other AUGs raising the question how the scanning ribosomal complexes could reach this AUG. In the absence of experimental evidence and plausible mechanisms explaining translation initiation at this AUG we did not consider it in our study. In all GENCODE transcripts AUG3 is annotated as the sole initiation site (Fig. 1a).

### Aggregated ribosome profiling data suggest predominant translation at AUG1

Ribosome profiling data aggregated in GWIPS-viz [18], Trips-Viz [17] and RiboCrypt [26] confirmed expression of the AUG3 initiated translon (translated region [27] encoding the major SRD5A1 proteoform as well as that of the AUG2 initiated translon. Figure 1b shows a screenshot of aggregated data from 2783 datasets available in RiboCrypt [26]. The data indicate that the translation initiated at AUG1 is predominant as the majority of footprints support its reading frame (green) from its start to its stop (segments 1–4 in Fig. 1c). Translation initiation at AUG3 is also evident since footprints downstream of the AUG1 initiated translon (non-overlapping segment 5 in Fig. 1c) predominantly support its frame (blue). Detection of AUG2 initiated translon is the hardest because it overlaps with the other two near-entirely. However, the data do support its translation because footprints in the area where all three translons overlap (segment 3 in Fig. 1c) have higher support for its frame than for that of AUG3 (red over blue), while downstream of the AUG2 translon (segment 4 in Fig. 1c) footprints supporting AUG3 frame dominate over the AUG2 frame (blue over red). This switch likely occurs because of termination of ribosomes translating AUG2 translon (red). Figure 1d provides a nucleotide resolution of footprint density for the area of three AUG codons which is also consistent with usage of all three AUGs as start codons. It can be seen that the highest peaks of inferred P-site codons at these AUG codons match their respective frames.

Publicly available ribosome profiling data demonstrated that all three AUG codons serve to initiate translation in three overlapping frames in the *SRD5A1* 5’ leader (Fig. 1a-e). AUG1 is in a poor Kozak context (uauAUGu) which supports efficient leaky scanning allowing for the downstream AUGs to be initiated. Most current transcript annotations indicate AUG3 as the sole translation initiation site. In transcripts NM_001324322 and NM_001324323, AUG2 and an AUG from the second exon are annotated as initiation sites, respectively, the rationale most likely being that initiation at these codons would support the synthesis of the longest and catalytically active proteoforms from each transcript.

However, since all transcript isoforms share exon 1, and there is no evidence that the selection of start codons by the ribosomes depend on the exons organization downstream, the scanning ribosome will be similarly likely to initiate at an AUG codon in each of the transcript isoforms. The resulting products of translation will depend on the downstream exon organisation. To illustrate the transcript specific translation paths, we took advantage of Ribosome Decision Graphs (RDGs) [28] (Fig. 1f, g). Informed by our experimental data, RDG representations predict the synthesis of at least three distinct polypeptides from each transcript isoform.

Translation machinery varies across cell types [29] and conditions, it is particularly sensitive to stress [30, 31]. To examine whether relative expression of the AUG1-, AUG2- and AUG3 initiated translons changes across cells and conditions we analysed 2783 Ribo-seq libraries (Additional file 1: Table S1) and clustered them based on relative translation of 5 segments depicted in Fig. 1c (Methods). This led to the identification of five distinct data clusters (Fig. 1e and Additional file 2: Fig. S1). Cluster 5 is characterised by elevated relative translation of the AUG3 initiated translon. This cluster comprises of 29 Ribo-seq libraries derived from 8 studies representing three cell lines: HeLa (12 samples), PC3 (7 samples), MCF (4 samples) (Additional file 1: Table S1). The remaining two samples are from human liver tissue (Additional file 1: Table S1). Of note, all 7 PC3 libraries in cluster 5 originated from the same study [32]. In our dataset there is only one other study featuring PC3 cells [33] however the coverage was insufficient and did not pass our threshold for expression levels. The enhanced AUG3 translon expression observed in cluster 5 (samples from breast, ovary and prostate cancer cell lines) is consistent with previously observed elevated SRD5A1 activity in sex hormone related cancers [21–23].

In addition to analysing distribution of footprints of elongating ribosomes we took advantage of the data obtained with translation inhibitors that selectively arrest initiating ribosomes [34–36]. This type of data is less quantitatively reliable due to reliance on discrete locations of potential start codons making them prone to sequence biases. Yet, it may be used for qualitative assessments of translation initiation. Out of the 8 initiation studies available in the GWIPS-viz browser, 4 had footprint signals above background levels in the *SRD5A1* 5’ leader, and from these, preferential initiation at AUG1 was revealed for MCF, HCT116 and iPSC cells (Additional file 2: Fig. S2). Only one study, from Jurkat cells, had the largest initiation peak corresponding to initiation at AUG3, providing additional evidence for cell type specific effects on start site selection. Interestingly, translational effects specific to Jurkat cells have been noted previously [37].

### Reporter evidence for triple decoding in the SRD5A1 5’ leader

**Fig. 2:**
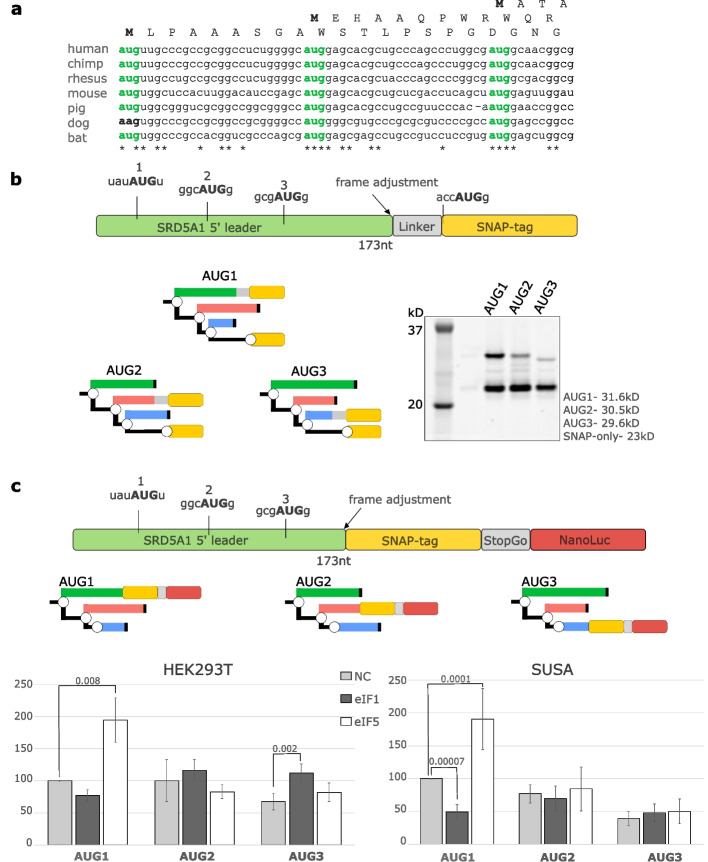
Reporter assay validation of translation initiation at three AUG codons in the *SRD5A1* 5' leader. **a** An alignment of representative mammalian genomic sequences in the vicinity of the 3 AUGs in *SRD5A1* 5’ leader showing examples of AUG losses. **b** SNAP-tag reporter assay. A schematic of reporters where SNAP encoding sequence was fused downstream of the *SRD5A1* 173 nt 5’ leader/CDS sequence is shown on top with RDG representations of individual constructs shown below. The frame adjustment allows the SNAP reading frame to be in frame with AUG1, AUG2 or AUG3 in the three constructs. **c** Luciferase reporters assay measuring the effect of eIF1/eIF5 overexpression on AUG selection in HEK 293 T and SUSA cell lines. A schematic of the construct where NanoLuc activity reports on each of three reading frames expression is at the top with individual constructs represented as RDGs below. The bars represent normalised NanoLuc activities in cells expressing eIF1 (dark gray), eIF5 (light gray) and negative control (NC, white) from 3 independent experiments (each with 4 technical replicates) calculated as percentage of AUG1 NC. Two-tailed Student's t-test with equal variance and with significance threshold set at *p* < 0.05 was applied to assess the significance of the difference of eIF1/eIF5 to NC for each AUG

The nucleotide sequence in the vicinity of the three AUG codons is shown in Fig. 2a along with sequences of other mammals. To examine AUG utilisation by an orthogonal approach, we generated reporter constructs where the full-length 5’ leader sequence of the canonical *SRD5A1* transcript (ENST00000274192.7/NM_001047), followed by the first ten codons downstream of AUG3, were fused upstream of the SNAP-tag encoding sequence (Fig. 2b). Initially, three *SRD5A1*-SNAP constructs were generated, with the SNAP-tag encoded in each of the three reading frames. Detection of SNAP-tag-containing products following transient transfection of HEK 293 T cells confirmed the evidence from ribosome profiling data, namely that ribosomes initiate translation at all three AUG codons (Fig. 2b). Moreover, the intensity of the bands suggested that AUG1 was the most efficiently utilized, followed by AUG2 and AUG3. Note that the relative abundance of the bands can be affected by differential stability of the three SNAP-fusion products.

The occurrence of a SNAP-only band of approximately 23 kD (Fig. 2b) most likely resulting from initiation at the SNAP-tag AUG in a perfect Kozak context [38, 39], suggested a highly efficient leaky scanning of the *SRD5A1* 5’ leader. However, it would be misleading to assume that the high intensity of this SNAP-only band solely reflects the number of ribosomes reaching the SNAP AUG codon. It is likely that the SNAP-only product is more stable or exhibits a greater affinity for the SNAP-tag substrate compared to the N-terminally extended proteoforms [40].

To validate that the observed bands originated from initiation at each of the three AUG codons, specific nucleotide substitutions were introduced (Additional file 2: Fig. S3). Changing each AUG codon to an AAG resulted in a complete loss (for AUG1 and AUG2) or substantial reduction (for AUG3) of the corresponding band on the gel (Additional file 2: Fig. S3). Mutating AUG1 to AAG led to an expected increase in initiation at the two downstream AUG codons, while initiation at AUG3 was also enhanced by AUG2 to AAG substitution (Additional file 2: Fig. S3). These findings imply that, as predicted by the leaky scanning model of translation, the expression of AUG3 could be regulated by modifying the efficiency of initiation at the upstream AUGs. Placing AUG1 in an optimal Kozak context eliminated the band corresponding to initiation at AUG2, while initiation at AUG3 was still detectable arguing that some scanning ribosomes continue through the first two AUGs even when these are placed in an optimal context (Additional file 2: Fig. S3).

### Stringency of start codon selection alters relative translation of three translons

Initiation efficiency of start codons, particularly of those with sub-optimal Kozak context such as AUG1, is dynamically regulated by the levels of specific translation factors, such as the stringency factors eIF1 and eIF5 [41, 42]. It was shown that the levels of these and other translation factors vary between different cell types, thereby creating distinct cellular environments affecting translation [29]. We compared the *SRD5A1* start codon utilization in HEK 293 T and the testicular germ cell SUSA upon overexpression of eIF1 and eIF5. To this end we generated constructs where NanoLuc activity reports on translation of each frame. In these constructs, NanoLuc was fused to SNAP-tag (Fig. 2c and Methods). These constructs lack both the linker sequence and the perfect AUG codon just 5’ of the SNAP-tag coding sequence. Placing the StopGo (also known as 2 A) [43, 44] sequence between that of SNAP-tag and NanoLuc aimed to neutralize the effects of different N-termini on NanoLuc activity.

We examined the NanoLuc reporter constructs in cells co-transfected with eIF1 or eIF5 expressing vectors (Fig. 2c). Consistent with its role in enhancing the stringency of start site selection [42], elevated levels of eIF1 led to a reduced initiation at AUG1 which is in a poor Kozak context. This effect was more pronounced in the testicular germ cell line SUSA possibly reflecting differences in endogenous eIF1 levels. A nearly two-fold increase in AUG1 expression was observed upon overexpression of eIF5 which reduces stringency of start site selection. Initiation at AUG2 and AUG3, both in near optimal Kozak context, was less significantly affected; most notably, eIF1 overexpression in HEK 293 T cells resulted in an approximately two-fold increase in AUG3-initiated translation.

### Triple decoding protects protein expression from frameshift mutations

**Fig. 3:**
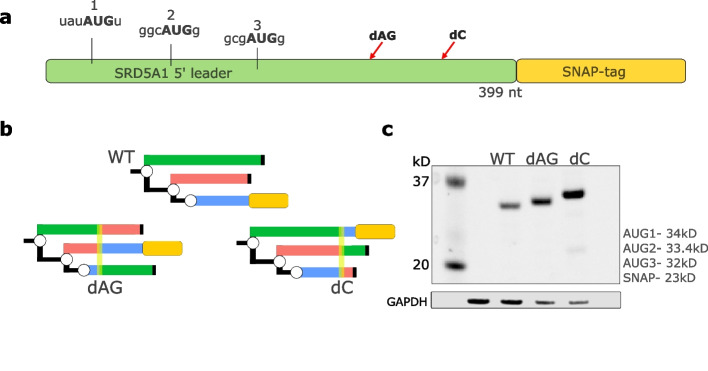
Overlapping translation in *SRD5A1* provides a safeguard mechanism against the effects of indel mutations. **a** A schematic of the SNAP reporter constructs where SNAP is fused downstream of *SRD5A1* 399 nt 5’ leader/CDS sequence. In addition to the reference sequence (WT), two mutant constructs were generated harbouring one of two naturally occurring indel variants, dAG and dC respectively. **b** RDG representations for the three constructs. The parts of individual translons are coloured according to reference sequence, thus a change of colour indicates a change in reading frame caused by frameshift variants. **c** SNAP-tag reporter assay of the constructs containing reference and variant *SRD5A1* sequences

Our observation that a significant portion of the coding region is translated in all three reading frames has important implications for the understanding of the effects of an indel mutation occurring within the overlapping region. We predict that indel mutations typically causing frameshifts and preventing protein expression due to premature termination and subsequent nonsense-mediated decay [45] would not have the same deleterious effect in *SRD5A1*. Instead, we expect persistent expression of a proteoform containing the SRD5A1 catalytic subunit, but with alternative N-terminus that may or may not affect its activity. To test this hypothesis experimentally, we assessed the effects of two naturally occurring SNPs on SNAP expression: an AG deletion at position 245 nt from the 5’ end (dbSNP rs1489726635) causing a −2 frameshift, and a C deletion at position 363 nt from the 5’ end (dbSNP rs1314274814) causing a −1 frameshift. We extended the *SRD5A1* test sequence from 173 to 399 nts to include these SNP positions (Fig. 3a, b). Additionally, we removed the linker and the SNAP accAUGg codon to eliminate any ambiguity regarding the origin of observed bands on the gel (Fig. 3). This modified construct is referred to as the wild type (WT). We then generated two additional constructs containing either the dAG or dC SNPs, without applying frame adjustments to any of these three constructs. As predicted, SNAP was expressed in the wild type as well as in both constructs containing frameshift mutations (Fig. 3c). These results suggest a remarkable safeguard mechanism against the deleterious effects of frameshift mutations and necessitate an updated approach to interpreting genomic variance.

### Phylogenetic analysis and reanalysis of mass spectrometry proteomics datasets

Comparative sequence analysis provided support for the ENST00000274192.7/NM_001047 transcript being the ancestral form, at least with respect to the mammalian common ancestor. All three AUGs are likely ancestral to placental mammals, see genomic sequence alignments in Fig. 2a and Additional file 2: Fig. S4. From an evolutionary perspective, it is striking that the CDS in the first exon of this transcript is highly divergent within mammals, especially over the first ~ 50 aa. Even just between human and mouse this region is hard to align suggesting that the protein is highly dynamic in evolutionary terms. This would seem to fit with the overall narrative about the function of the gene *SRD5A1* being involved in testosterone processes, with the role of testosterone in mammals itself apparently being subject to rapid changes [46].

Although all three AUG codons seem ancestral, independent losses of AUG1 and AUG2 are observed in multiple lineages in placental mammals (Fig. 2a and Additional file 2: Fig. S4). Furthermore, stop codons interrupting their ORFs (relative to human) are even more frequent across different species, suggesting weak selection (if any) on the products of their translation initiation. Given the comparatively high conservation of these AUGs and the knowledge that AUGs in 5’ leaders are generally under negative selection [47, 48], it is reasonable to infer their functionality. To gain further insight into potential biological role of non-catalytic proteoforms encoded by AUG1 initiated translon we performed the reanalysis of publicly available mass spectrometry proteomics data (Methods and Additional file 2: Table S2). We found high confidence peptides matching the catalytic proteoforms, but none matching AUG1 initiated proteoforms (Fig. 1g and Additional file 2: Table S3). This suggests that these proteoforms are highly unstable and are not present at significant levels in the cell despite much higher synthesis level in comparison with the catalytic proteoforms. Indeed, it has been observed that most annotated functional proteins have different amino acid composition from those generated during translation of translons not coding for proteins affecting their cellular stability [49].

Given these results, i.e. evidence of evolutionary selection acting on AUG1, but not on the product of its translon combined with the lack of mass spectrometry support, it is very likely that the biological significance of AUG1 and its translons lies in the regulation of SRD5A1 catalytic proteoform expression rather than in the products of their translation.

### Frequency of triple coding in humans

**Fig. 4:**
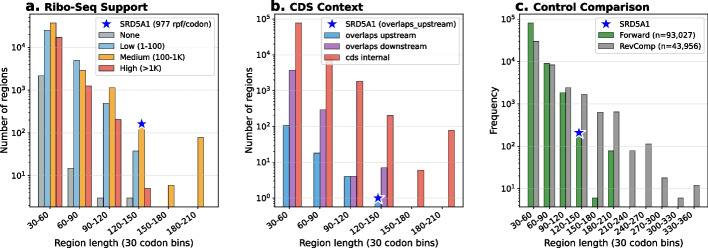
Distribution of triple-frame overlaps between ATG-Stop ORFs. **a** Ribosome footprint coverage. Frequency of overlapping regions is shown for different lengths (bins of 30 codons) depending on the number of ribosome footprints as indicated within the figure. Blue asterisk shows position of *SRD5A1*. **b** Same as **a** but for overlaps depending on their orientation relative to CDS, internal if the overlap is within CDS, upstream if it starts with CDS start and downstream if it ends with CDS stop. **c** Distribution of ORFs overlaps in three reading frames in the annotated transcripts (green) compared to their distribution in the reverse complement sequences (grey)

Intrigued by the case of > 100 codons translation of all three reading frames in the region of *SRD5A1* locus, and prompted by a suggestion from an anonymous reviewer, we have decided to explore how frequent this phenomenon might be elsewhere in the human genome. For this purpose, we have analysed how frequently overlaps between ORFs occur in all reading frames in the annotated transcripts using Gencode catalogue v45 [50]. The ORFs were defined either as the longest sequence of codons not containing stop codons between ATG and a stop codon or between two stop codons according to the two most common definitions of ORFs [27, 51], see Methods. The detailed information on regions overlapping in all three reading frames is provided for ATG-stop ORFs (Additional file 3: Table S4) and stop-stop ORFs (Additional file 4: Table S5) along with information on the number of ribosome footprints aligning to these regions in RiboCrypt aggregated data, see Methods. To obtain an estimate of chance occurrence of triple overlapping regions we also carried this analysis on ORFs of reversed strands. The frequency of overlapping regions of different lengths is shown in Fig. 4 and Additional file 2: Fig. S5. Their distribution approximately follows power law distribution typical for other naturally occurring sequence patterns, such as k-mer repeats [52]. Therefore, there is a large number of very short overlaps, while > 100 codons overlaps are extremely rare. We found them in the transcripts deriving from only 45 gene loci (Additional file 3: Table S4). The distribution of triple-frame overlaps on the reverse strand is very similar to that on the positive strand, however, the exceptionally long overlaps seem to be avoided on the positive strands in comparison with the negative strand Fig. 4c and Additional file 2: Fig. S5c which may suggest negative evolutionary selection on long overlaps in accordance with Ambush Hypothesis [53]. It argues for the existence of evolutionary selection for stop codons in reading frames alternative to CDS frame that prevents synthesis of long trans-frame polypeptides due to accidental ribosomal frameshifting [53].

Overlaps between ORFs are not necessarily translated in all three frames, since most ORFs are not translons [27]. Detecting overlapping translation automatically is not a trivial task. As shown earlier even detection of overlapping translation in two reading frames is highly dependent on the length, ribosome profiling coverage and relative translation of overlapping translons [8]. Triple coding in the *SRD5A1* loci was detected serendipitously because it already contained annotated CDS in two reading frames and ribosome profiling was overwhelmingly supporting translation of the third frame. To confirm triple coding we relied on the analysis of framing bias with different combinations of overlapping regions (Fig. 1c) where changes in reading frame bias can be matched to changes in overlapping status of corresponding segments. Translation of three reading frames with roughly the same translation efficiency would be expected to exhibit no framing bias. However, the lack of framing bias may also be observed due to footprints generated by the protection from RNA-binding proteins or nucleoproteins other than the ribosome and therefore cannot be considered as a signature of genuine translation. The analysis such as shown in Fig. 1c requires segmentation of overlapping regions and partly or non-overlapping regions of overlapping ORFs. In the absence of computational approaches for such segmentation and corresponding framing bias analysis we carried out this task manually for the loci where triple overlaps exceed 100 codons and where the coverage of ribosome profiling in RiboCrypt exceeds 10 K mapped reads (for the reference *SRD5A1* coverage is > 120 K). Out of 31 candidates that passed these filters only SRD5A1 and one additional locus (*HEXD*) exhibited evidence of three translons within the triple overlapping region and two more *ABHD8* and *PDE2A* show evidence of two translons, i.e. dual coding, albeit *PDE2A* exhibits translation also in the third frame, but outside of the triple overlapping region (Additional file 2: Fig. S6). A large number of alternatively spliced transcripts originate with *HEXD* and *PDE2A* loci. Based on this and sequence organisation of these two loci it is very likely that in these genes the overlapping regions are translated in different frames on alternative transcripts rather than on the same transcript as in *SRD5A1* and *ABHD8* mRNAs. The translation of overlapping translons in *HEXD* and *PDE2A* mRNAs is seen in ribosome profiles (Additional file 2: Fig. S6) because footprints derived from one transcript map to all other transcript isoforms that share the corresponding sequence akin to previously reported dual coding in *PHPT1* and *c11orf48* [8].

Thus, so far, based on the available ribosome profiling data, *SRD5A1* seems to be a unique human locus where three translons overlap in all three reading frames within the same transcript for over 100 codons. However, this does not mean that *SRD5A1* is unique. There is a large number of shorter overlaps that we have not been able to analyse manually due to their large number. Also, it is possible that some of the transcripts with overlapping ORFs have insufficient coverage for this analysis among available datasets. It is also conceivable that some translons are condition specific, e.g. as dual coding translation that was reported for *ROMO1* gene only under conditions of severe oxygen and glucose deprivation [30].

## Discussion

We present the first documented case of triple decoding in a human gene, where three long overlapping open reading frames are translated from the same mRNA at a region over 300 nt, a phenomenon previously considered only a theoretical possibility. These translons result in the synthesis of at least three different polypeptides from each *SRD5A1* mRNA, as illustrated with ribosome decision graphs (Fig. 1g). Only two out of the nine proteoforms (initiated at AUG2 or AUG3 depending on the transcript) contain a catalytical domain of steroid 5-alpha-reductase, while the biological significance of the other seven proteoforms remains unclear.

Most surprisingly, both ribosome profiling and expression reporter evidence indicate that AUG1 is by far the most efficient initiation codon, despite not producing proteoforms with a catalytic domain from any of the annotated transcripts. Given that we have not been able to detect peptides matching these proteoforms in available public mass spectrometry proteomics datasets and the absence of evidence for evolutionary purifying selection, the AUG1-initiated polypeptides most likely rapidly degrade in the cell and very likely have no biological roles beyond potentially contributing to the pool of immunopeptides.

Nonetheless, our data suggest that the translation of AUG1 translons modulates the synthesis of catalytic proteoforms, as we observed differential translation of the same RNA reporters in different cell lines (HEK 293 T and SUSA). Additionally, we demonstrated that altering the stringency of start codon selection can influence the relative synthesis of these proteoforms. Since AUG1 is in a poor Kozak context, reduced stringency increases its efficiency, thereby repressing the synthesis of catalytic proteoforms initiated from downstream AUGs. By analysing thousands of available ribosome profiling datasets, we have also identified differential translation of these translons across various human samples. This variation may be due to differences in the abundance of translation factors or the composition of RNA-binding proteins interacting with *SRD5A1* mRNA. Many different translation factors influence stringency of start codon selection, reviewed in [54], and their concentrations vary across cell lines [29]. Moreover, it has also been shown that eIF1 cytoplasmic concentration varies across cell cycle due to its release from nucleus during mitosis [55]. Our study highlights the utility of large-scale Ribo-seq data reanalysis in characterizing differential translation in varying cellular environments.

The surprising finding that the unannotated and apparently non-functional polypeptide is synthesized at the highest level raises questions for future genome annotation efforts. To date, annotation has focused on identifying protein-encoding sequences under purifying selection using codon substitution models [56] as the gold standard. Purifying selection acts on specific genotypes due to their positive effects on fitness, unambiguously indicating the functional significance of the encoded feature. However, the advent of ribosome profiling, which provides an unbiased assessment of protein synthesis at the whole-cell level, has revealed widespread translation beyond the regions annotated as protein-coding [57, 58]. The significance of these enigmatic translons, also known as RiboSeq ORFs, is currently under investigation [59]. It is likely that a substantial fraction of these translons arises due to mechanistic requirements of the translation initiation process rather than adaptive evolution, explaining the lack of evolutionary selection acting on their product sequences. Nonetheless, they result in the production of a significant number of polypeptides that may potentially affect biochemical processes in our cells, with either positive or negative consequences. The example of *SRD5A1* illustrates that, in certain loci, the translation of such enigmatic translons could exceed the translation of those encoding proteins under evolutionary selection. While its products are unstable and subject to rapid degradation under normal conditions, these polypeptides may become particularly relevant when cellular proteostasis mechanisms are compromised or overwhelmed.

Therefore, our findings emphasise the need to annotate productive translation irrespective of the functional role of its products. This is highly relevant for the appropriate interpretation of genomic sequence variants. Currently, it is assumed that an indel mutation occurring in a protein coding region would be deleterious for expression and function due to premature termination in the shifted reading frame. The exact position of an indel within CDS is taken into account during the interpretation of the indel as a deleterious frameshift mutation [60]. When they occur at the last exon or at the end of the penultimate exon, the resultant premature stop codon is unlikely to trigger nonsense-mediated decay [45]. As a result, protein expression persists, albeit with a different C-terminus because of the frameshift. The different C-termini may or may not significantly affect the activity of the protein, and therefore, it is possible that such indels would not have a deleterious effect. Here we report another consideration, specifically whether CDS of a protein overlaps with other translons in alternative reading frames. An indel in such an overlapping region could result in persistent synthesis of the proteoform containing the same CDS-encoded protein sequence downstream, but with a different N-terminus that may or may not significantly affect the protein activity. We have demonstrated that this is the case using genetic reporters. An indel mutation within the overlapping region of three translons is not deleterious for reporter production. In the case of SRD5A1 expression, this means that its main catalytically functional proteoform will be synthesized in the presence of such mutations, albeit with alterations to its N-terminus that may worsen its activity and stability. Nonetheless, it will always be produced from one of the three AUG codons in any frame. Moreover, a single nucleotide deletion would place the catalytic domain in-frame with AUG1, thus likely increasing the levels of its synthesis.

Finally, this finding is also relevant to design of gene knockout screens. The resistance of gene expression to indels in coding regions is not limited to triple decoding which is rare. Either a deletion or insertion of a nucleotide is expected to retain gene expression in cases of dual decoding, which is common.

## Conclusions

*SRD5A1* locus contains a long segment translated in all three reading frames into three long polypeptides. This organisation of protein coding information allows for differential regulation of translation in different cellular environments and also provides resistance of SRD5A1 catalytical domain expression to indel mutations in the region where the three translons overlap. The indels, however, will result in the change of N-terminus amino acids composition that may still impair the activity and stability of the protein. Nonetheless we show that protein expression may persist in the presence of indels occurring in overlapping regions. This should be taken into account when interpreting frameshift causing indels. These considerations argue for the need to account for all translons, irrespective of the functional roles of their translation or their polypeptide products.

## Methods

### Ribosome profiling data analysis

Per-sample (2783 libraries) RiboCrypt P-site coverage tracks for *SRD5A1* locus were used for the clustering and translation detection evidence (Fig. 1b-e and Additional file 2: Fig. S1). Clustering of entire transcript-level coverages was performed with the unsupervised k-means algorithm into 5 clusters, a functionality available under RiboCrypt metabrowser utility [26]. Relative translation analysis of the three overlapping translons (Fig. 1c-e) was done by summing P-site coverage across all libraries for 5 unique overlaps between the 3 translons: Just translon 1, translon 1 + 2, translon 1 + 2 + 3, translon 2 + 3, translon 3.

### Mass spectrometry proteomics data analysis

Seven public label-free DDA (Data Dependent Acquisition) public mass spectrometry proteomics datasets were selected from the PRIDE database [61] for reanalysis, corresponding to the cell lines HEK 293 T and/or human tissue generated in baseline/healthy conditions, all of them generated from Thermo Fisher Scientific instruments. The datasets PXD002613, PXD003133, PXD019483 comprised samples from the HEK 293 cell line, while PXD020630 had samples from HEK-293 T cell line. Healthy tissue samples were selected from datasets PXD010154, PXD010271and PXD000561. All tissue samples were analysed from dataset PXD010154 and only liver tissue samples were analysed from datasets PXD010271 and PXD000561.

All datasets were analysed individually on a high-performance Linux cluster using MaxQuant [62] version 2.1.0.0 with default parameters. MS1 and MS2 tolerances were set to 20 ppm and 0.5 Da respectively. Trypsin was used as the digestion enzyme and 2 missed cleavage sites were allowed. Carbamidomethylation of Cysteine was set as fixed modification, while protein N-term acetylation and oxidation of Methionine were set as variable modifications. PSM (peptide spectrum match) and protein FDR (false discovery rate) levels were set at 1% and match between runs was set to FALSE. The human UniProt ‘one protein sequence per gene’ protein sequence database (20,602 sequences, downloaded on May 2023) (UP000005640_9606) was used as target sequence database. The 9 ORF sequences translated from all three reading frames of SRD5A1 were added to the target sequence database. The inbuilt MaxQuant contaminant database was used and a decoy database was generated using MaxQuant at the time of the analysis (on-the-fly) by reversing the input database sequences after the respective enzymatic cleavage.

### Phylogenetic analysis

Genomic sequence alignments were visualised and explored with CodAlignView [63] using 100-way vertebrate genomic alignment [64] with human genome hg38 assembly as a reference.

### Identification of all three frames overlaps

All RNA transcripts in the human transcriptome (GENCODE v45 [50]) were scanned for regions with overlapping ORFs in all three frames. ORFs shorter than 30 codons were discarded. To estimate the rate at which ORFs overlapping in all three frames occur in untranslated sequences the same analysis was performed on the reverse complement of each transcript. ORFs were defined either as the longest uninterrupted sequence of codons between ATG and a stop codon (ATG-Stop) or between two stop codons (Stop-Stop) [27, 51]. Each candidate region was categorised based on the overlapping segments position relative to its transcripts CDS. Categories include, ‘Includes CDS’ where the overlap is an exact match to the CDS, ‘Overlaps Upstream’ or ‘Overlaps Downstream’ where either CDS start or CDS stop (but not both) are a part of the overlap and ‘Internal’ if the overlap region does not contain start or stop of CDS. Overlapping regions that did not contain annotated CDS were devided into two categories. Regions from protein-coding mRNAs where the annotated CDS lay outside of the overlapping region were categorised as ‘No Overlap’. Regions from non-coding RNAs with no annotated CDS were categorised as ‘No Annotation’, see Additional file 3: Table S4 and Additional file 4: Table S5.

Ribosome profiling support for each overlapping region was assessed using coordinates of ribosome P-sites inferred from uniquely mapped ribosome profiling reads from RiboCrypt bigWig files [65]. Read coverage was projected onto transcript coordinates in a splice-aware manner using exon boundaries from the reference annotation and number of counts was scaled by region length measured in codons.

### Cloning and sequences

Primers were synthesized by IDT, and their sequences are listed in (Additional file 2: Table S6). The primers were designed to amplify the sequence of the entire *SRD5A1* 5’ leader (as in MANE transcript) until and including 10 codons downstream of AUG3. This sequence was generated by a polymerase chain reaction (PCR) using as a template genomic DNA extracted from HEK 293 T cells. The test sequence was cloned (SacI + XbaI) (New England Biolabs (NEB)) directly following that of the CMV promoter in pcDNA3.4 vector expressing SNAP-tag. The last 40 codons from hemoglobin beta (*HBB*) coding region were used as a linker between the *SRD5A1* sequence and that encoding SNAP-tag. Expression of the three reading frames was monitored in three separate constructs, where SNAP-tag was placed in alternative reading frames by inserting one or two nucleotides following the 5’ leader sequence. Mutations were generated using in vivo assembly (IVA) cloning as described in [66]. In the constructs used for the luciferase assay, StopGo-NanoLuc encoding sequence, obtained as gene block from IDT, (Additional file 5) was introduced in place of the linker-SNAP -tag cassette in *SRD5A1* SNAP-tag vectors.

### Tissue culture and cell transfection

Human Embryonic Kidney 293 T (HEK 293 T) cells (ATCC), were maintained as monolayer cultures, grown in Dulbecco’s modified Eagle’s medium (DMEM), (Sigma-Aldrich) supplemented with 10% foetal bovine serum (FBS), 1% penicillin–streptomycin and 1% L-glutamine (Thermo Fisher). Human Testicular Epitheloid Cells (SuSa) (DMSZ) were grown as adherent monolayer cultures in 80% RPMI (Thermo Fisher) media supplemented with 20% FBS, and antibiotics at 37 °C in an atmosphere of 5% CO2.

### Transfection with SRD5A1-SNAP-tag expressing plasmids

4 × 10^6^ HEK 293 T and SUSA cells were plated on 10–cm tissue culture dishes. After 24 h, the cells were detached with trypsin, suspended in fresh media, and transfected with Lipofectamine 2000 reagent (Invitrogen), using the one-day protocol in which suspended cells are added directly to the DNA complexes in 24-well plates. For each transfection, the following was added to each well: 200 ng plasmid DNA, 1.3 μL Lipofectamine 2000, and in 200 μL Opti-MEM (Thermo Fisher). Next, 2 × 10^5^ for HEK 293 T, U2OS, SUSA and 1 × 10^5^ for PC3 in 800 μL DMEM were added to the transfecting DNA complexes in each well. Transfected cells were incubated for 24 h at 37 °C in 5% CO2.

### Transfection with SRD5A1-NanoLuc expressing plasmids

Cells were detached with trypsin, suspended in fresh medium, and transfected in four replicates with Lipofectamine 2000 reagent (Invitrogen), using the 1-day protocol in which suspended cells are added directly to the DNA complexes in half-area 96-well plates. For each transfection the following was added to each well: 25 ng of plasmid DNA, 0.2 μl of Lipofectamine 2000 in 25 μl of OptiMem (Gibco). 4 × 10^4^ cells in 50 μl of DMEM were added to the transfecting DNA complexes in each well. Transfected cells were incubated at 37 C in 5% CO_2_ for 20 h and assayed using the Dual-Luciferase assay.

### Protein isolation and electrophoresis

24 h after transfection, cells were washed with 1 × PBS, and whole-cell lysates were prepared in PLB (Promega) buffer supplemented with SNAP-Cell 647-SiR fluorescent substrate (final concentration 0,01 μM; NEB) and DTT (final concentration 1 mM; Biosearch Technologies). Cell lysates were incubated for 30 min at room temperature with shaking. Lysates were then clarified by centrifugation for 10 min at 16,000 × g at 4 °C and protein concentrations were measured using the Nanodrop Spectrophotometer (Thermo Fisher). Sample buffer (Bio-Rad Laboratories) supplemented with 5% β-mercaptoethanol was added to all protein lysates and samples were loaded onto a 4–12% Bolt™ Bis–Tris Plus Mini Protein Gel (Thermo Fisher). Protein gels were scanned with Typhoon Trio + instrument (Amersham) using the 670 BP 30 emission filter.

### Western blotting analysis

Proteins were separated by gradient SDS-PAGE using 4–20% polyacrylamide gels and transferred to a nitrocellulose membrane (Bio-Rad) using a Trans-Blot® SD Semi-Dry Transfer Cell (Bio-Rad) for 13 min at 2.5 A and 25 V. Membranes were blocked in 5% BSA in PBS-T for 1 h at room temperature. After blocking, the blot was incubated overnight at 4 °C on a rocker with a primary antibody against GAPDH (MA5—15,738, Thermo Fisher) diluted 1:5000 in 1% BSA in PBS-T. The membrane was then washed three times for 5 min each with PBS-T. A secondary antibody (Li-Cor 926−68022, IRDye 680LT) diluted 1:20,000 in 1% BSA in PBS-T was applied and incubated for 1 h at room temperature. Following three additional washes with PBS-T, the blot was visualized using a Li-Cor Odyssey imaging system.

### Dual-luciferase assay

The luciferase assay buffers were prepared in-house following the protocol described in [67]. Relative light units were measured on a Veritas microplate luminometer fitted with two injectors (Turner Biosystems, Sunnyvale, CA). Transfected cells were then lysed in 18 μl of 1 × passive lysis buffer (PLB; Promega), and light emission was measured following injection of 50 μl of each luciferase substrate buffer. A firefly-expressing plasmid was co-transfected alongside each *SRD5A1*-NanoLuc plasmid and was used as a transfection control. For each data point NanoLuc activity was normalized relative to the firefly activity. For each cell line, three independent transfection experiments were performed each with 4 technical replicates. The four data points for each construct were averaged, and standard deviations calculated. Although we have not used reporters encoded on the same RNA we followed recently developed MINDR guidelines [68] and report absolute readout values (Additional file 6: Table S7) and list the construct sequences (Additional file 5).

## Supplementary Information

Additional file 1: Table S1. Ribosome profiling datasets analysed in this study and their clustering based on the shape of SRD5A1 ribosome profiles.

Additional file 2: Figures S1-S7 and Tables S2, S3, S6.

Additional file 3: Table S4. Detailed information on regions overlapping in all three reading frames for ATG-stop ORFs.

Additional file 4: Table S5. Detailed information on regions overlapping in all three reading frames for stop-stop ORFs.

Additional file 5. Sequences of all constructs used in this study in FASTA format.

Additional file 6: Table S7. Raw data for absolute luciferase reporter readouts.

Additional file 7. Raw images of the gels used to design the figures.

## Data Availability

All data generated in this study and accession numbers for all publicly available data analysed in this study are included in this published article [and its supplementary information files]. The source code for computer scripts used in this study is available in GitHub at https://github.com/JackCurragh/SRD5A1 [69].
