# Supplementary material for: Human SRD5A1 as a case of gene expression indel-resistance in triple-coding region
Source: Genome Biol. 2026 May 18;27:219. doi: 10.1186/s13059-026-04106-x (PMC13352861; doi:10.1186/s13059-026-04106-x)
Supplement: Supplementary file 2 — Additional file 2: Figures S1-S7 and Tables S2, S3, S6. [file 13059_2026_4106_MOESM2_ESM.pdf]

Supplementary Figures S1-S6 and Supplementary Tables S2, S3 and S6.

for

Human *SRD5A1* as a case of gene expression indel-resistance in triple-coding region.

By

Martina M. Yordanova[1]*, Jack A. S. Tierney[1,2], Kyle A. Meiklejohn[1], Conor Slattery[1], Michał I. Świrski[3], Mirriam Baranova-Gurvich[1], Manon Engels[1], Oscar Ting[1], Ananth Prakash[2], Håkon Tjeldnes[1], Jonathan M. Mudge[2], Gary Loughran[1], Juan Antonio Vizcaíno[2], Dmitry E. Andreev[4,5], Pavel V. Baranov[1]*.

Table of content:

	pages
**Figure S1**	**2**
**Figure S2**	**3**
**Figure S3**	**4**
**Figure S4**	**5**
**Figure S5**	**6**
**Figure S6**	**7**
**Table S2**	**8**
**Table S3**	**9**
**Table S6**	**10**

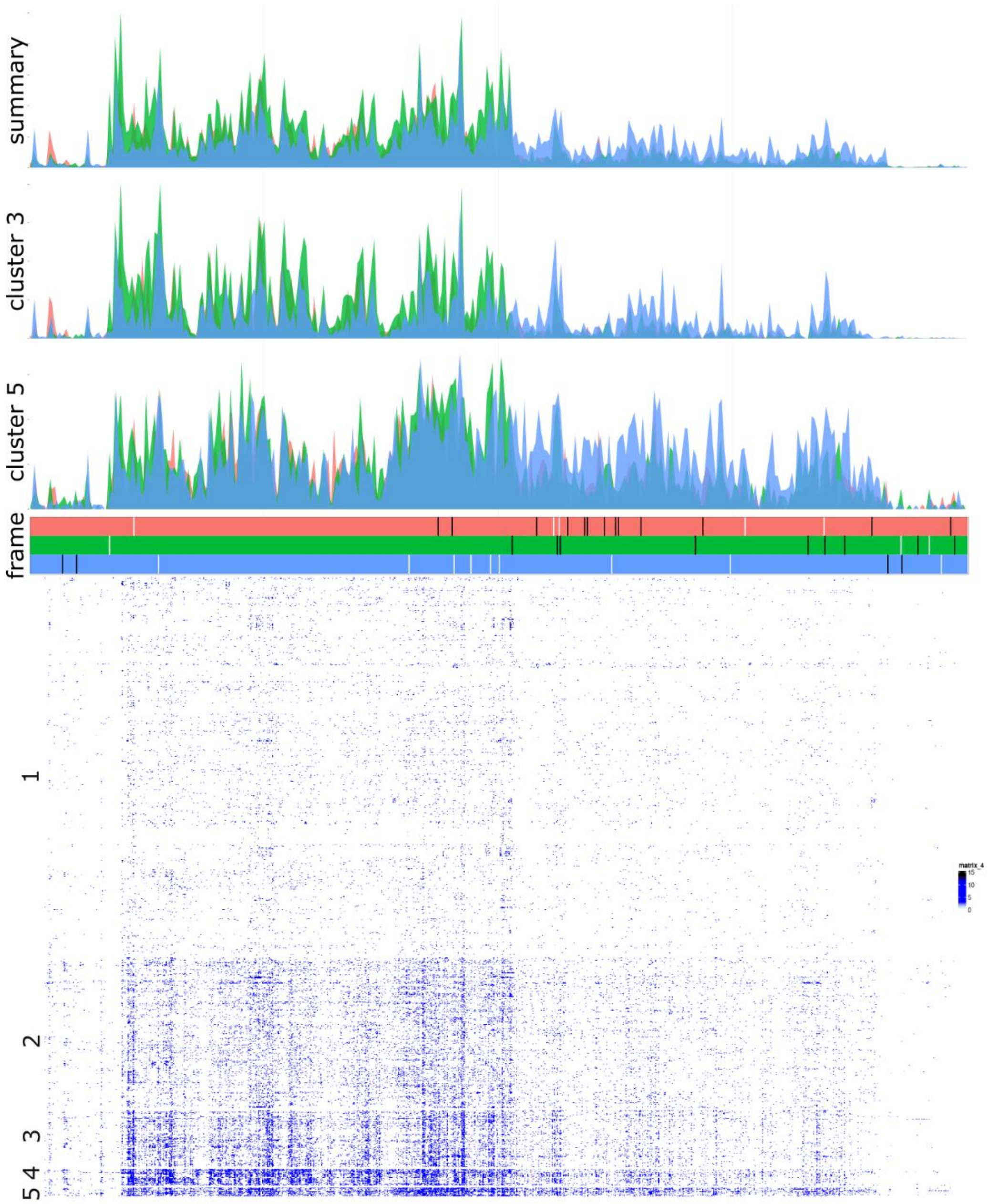

Figure S1. Analysis of aggregated Ribo-seq data identifies distinct clusters with differential ORF translation. Bottom: coverage heatmap of 2783 Ribo-seq libraries, grouped into 5 clusters with k-means algorithm. **Top**: sub-codon coverage plots for clusters 3, 5 and all samples combined showing differential ORF translation between clusters.

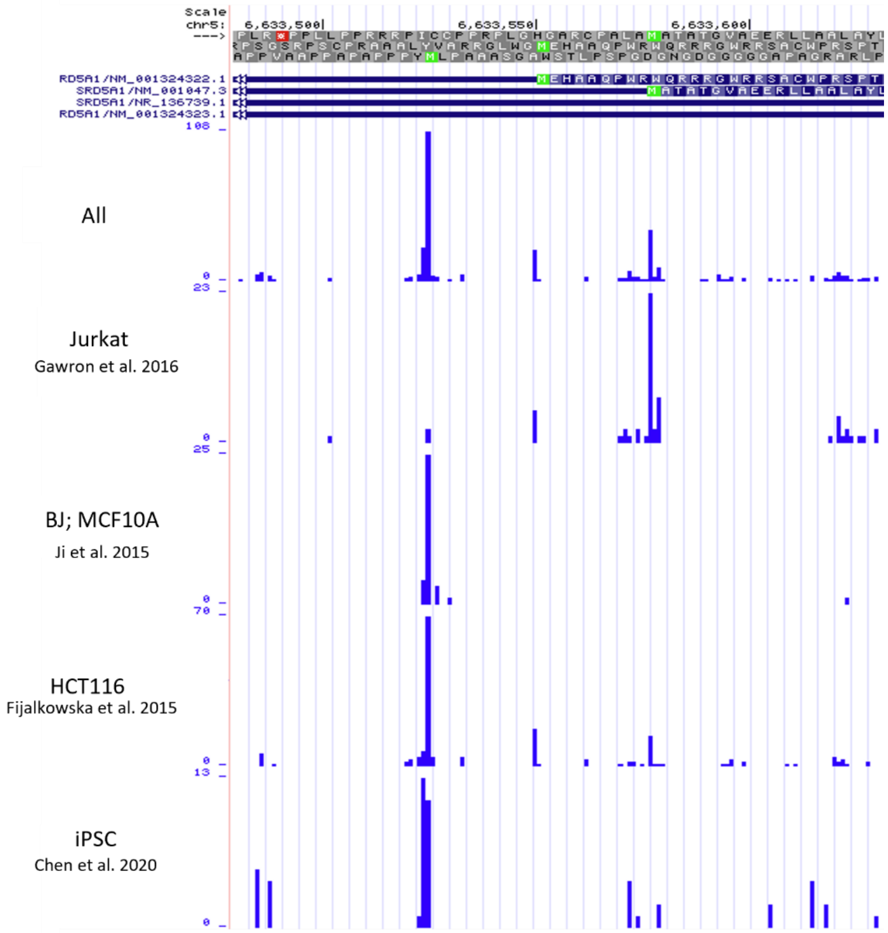

Figure S2. Ribosome footprints derived from Ribo-seq experiments that preferably select for initiating ribosomes. The frame plot shows the amino acid sequence in the three frames. The Met residues corresponding the three AUG codons are highlighted in green. Below are the RefSeq transcript annotations. Shown are data from all studies combined (upper row) and that from 4 individual studies, in Jurkat, BJ and MCF10A, HCT116, and iPSC. The first author and the year of study publication are indicated.

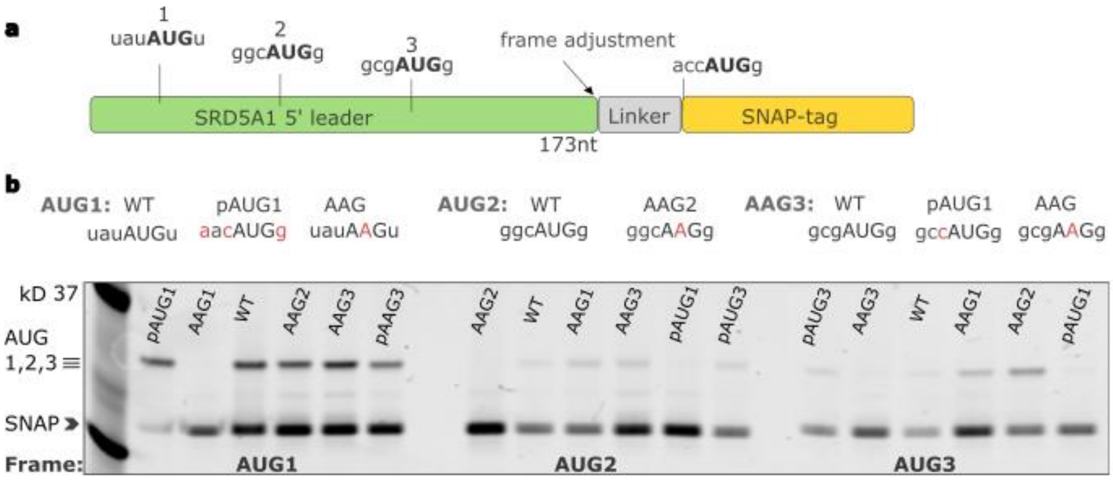

Figure S3. Site directed mutagenesis to validate the AUG codons used for initiation in the *SRD5A1* leader. a Schematic of reporters where SNAP encoding sequence was fused downstream to the *SRD5A1* 5' leader/CDS sequence. **b** Each of the three AUG codons was changed to an AAG; in addition, nucleotide changes were introduced to optimise the Kozak context of AUG1 and AUG3. The introduced nucleotide changes are in red font. The results from the WT and mutant SNAP reporter constructs are shown below; the monitored frame (AUG1, 2, 3) is indicated at the bottom of the SNAP gel.

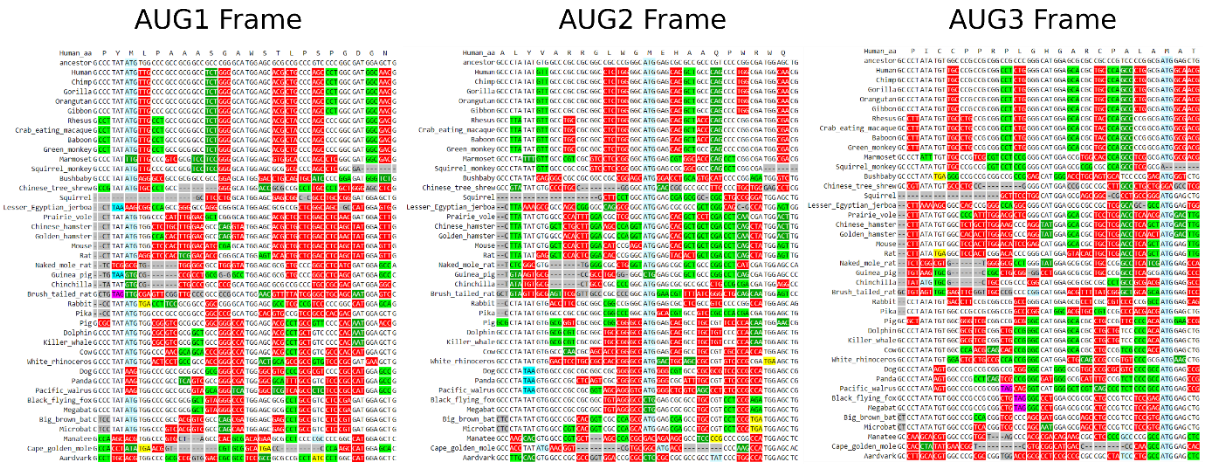

Figure S4. CodAlignView visualisation of 100-way vertebrate genomic alignment in the vicinity of *SRD5A1* AUG codons. Each panel corresponds to the reading frame matching one of the three AUG codons with AUG codons highlighted in blue, radical codon substitutions in red and synonymous or similar codon substitutions in light or dark green respectively. The first row shows the ancestral sequence predicted with CodAlignView.

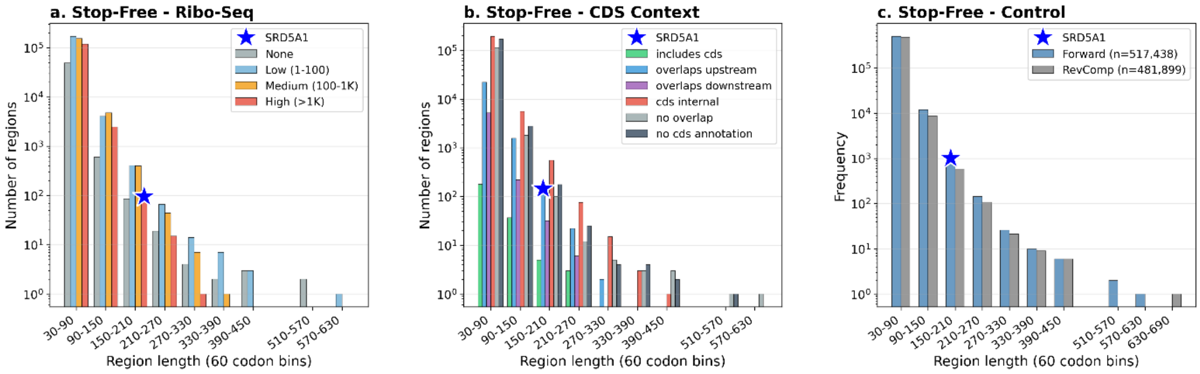

Figure S5. Distribution of three frames overlaps between Stop-Stop ORFs. a. Ribosome footprint coverage. Frequency of overlapping regions is shown for different lengths (bins of 20 codons) depending on the number of ribosome footprints as indicated within the figure. Blue asterisk shows position of *SRD5A1*. **b**. Same as **a** but for overlaps depending on their orientation relative to CDS, internal if the overlap is within CDS, upstream if it contains CDS start and downstream if it ends with CDS stop. **c**. Distribution of ORFs overlaps in three reading frames in the annotated transcripts (green) compared to their distribution in the reverse complement sequences (grey).

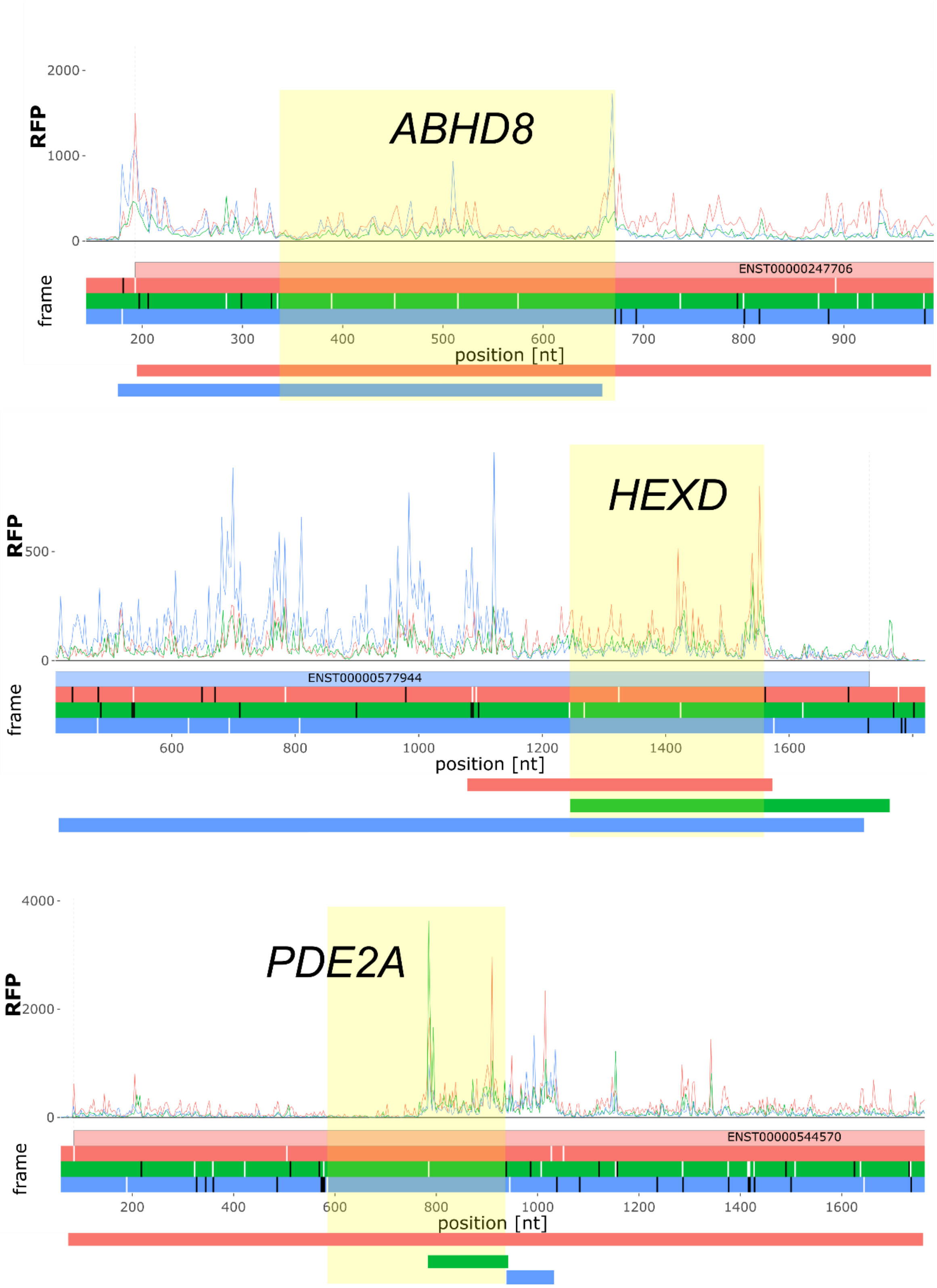

Fig. S6. Translation of alternative reading frames in triple overlaping regions. Ribocrypt screenshots of ribosome profiling densities for for *ABHD8*, *HEXD* and *PDE2A* transcripts. Regions where three ORFs overlap in all three reading frames are highlighted in yellow. Regions corresponding to translons supported with ribosome profiling data are indicated with coloured bars undermeath matching the reading frames.

Table S2. Summary of proteomics datasets reanalysed. §after removing reverse decoys and contaminants, *samples from healthy liver tissues only.

PRIDE dataset identifier	Source	Mass spectrometer	Num. of MS runs	Fractionation	Num. of PSMs	Num. of unique peptides	Num. of protein groups§
PXD002613 [3]	HEK-293	Q Exactive	34	Yes	152,123	36,716	4,691
PXD003133 [4]	HEK-293	LTQ Orbitrap Velos	4	No	68,475	25,727	3,742
PXD019483 [5]	HEK-293	Q Exactive	8	Yes	187,700	82,074	8,119
PXD020630 [6]	HEK-293T	Q Exactive HF	4	No	154,125	32,586	5,058
PXD010154 [7]	Tissues	Orbitrap Fusion Lumos<br>Q Exactive Plus<br>Q Exactive HF	324	No	2,687,837	173,957	10,848
PXD010271 [8]*	Tissue	LTQ Orbitrap Velos	10	No	102,185	19,596	2,583
PXD000561 [9]*	Tissue	LTQ Orbitrap Elite<br>LTQ Orbitrap Velos	184	Yes	487,859	79,343	6,138

Table S3. Universal Spectrum Identifiers for the mass spectra of peptides of alternative coding isoforms from all three reading frames (TIS1, TIS2 and TIS3) as well as from the canonical protein (UniProt accession: P18405, S5A1_HUMAN, Ensembl gene *SRD5A1*). To visualise MS spectra, use the USI here: https://www.ebi.ac.uk/pride/archive/usi

PRIDE USI	Sequence	Length	Proteins	Dataset	Raw file	Tissue
mzspec:PXD010154:01226_C01_P012502_S00_N03_R1:scan:26989:CLIYPFLMR/2	CLIYPFLMR	9	lcl|ORF36|TIS3|NM_001047|5exons;sp|P18405|S5A1_HUMAN	PXD010154	01226_C01_P012502_S00_N03_R1	Liver
mzspec:PXD010154:01226_E04_P012502_S00_N29_R1:scan:11289:EHHEWYLR/3	EHHEWYLR	8	lcl|ORF36|TIS3|NM_001047|5exons;sp|P18405|S5A1_HUMAN;lcl|ORF1|TIS2|NM_001324322|4exons	PXD010154	01226_E04_P012502_S00_N29_R1	Liver
mzspec:PXD010154:01284_E04_P013188_B00_N29_R1:scan:14019:EHHEWYLR/3	EHHEWYLR	8	lcl|ORF36|TIS3|NM_001047|5exons;sp|P18405|S5A1_HUMAN;lcl|ORF1|TIS2|NM_001324322|4exons	PXD010154	01284_E04_P013188_B00_N29_R1	Small intestine
mzspec:PXD010154:01093_D03_P010748_S00_N20_R1:scan:5152:KFEEYPK/2	KFEEYPK	7	lcl|ORF36|TIS3|NM_001047|5exons;sp|P18405|S5A1_HUMAN;lcl|ORF1|TIS2|NM_001324322|4exons	PXD010154	01093_D03_P010748_S00_N20_R1	Liver
mzspec:PXD010154:01226_A05_P012502_S00_N33_R1:scan:20366:YLSHCAVYADDWVTDPR/3	YLSHCAVYADDWVTDPR	17	lcl|ORF36|TIS3|NM_001047|5exons;sp|P18405|S5A1_HUMAN	PXD010154	01226_A05_P012502_S00_N33_R1	Liver
mzspec:PXD010154:01226_H04_P012502_S00_N32_R1:scan:21475:YLSHCAVYADDWVTDPR/3	YLSHCAVYADDWVTDPR	17	lcl|ORF36|TIS3|NM_001047|5exons;sp|P18405|S5A1_HUMAN	PXD010154	01226_H04_P012502_S00_N32_R1	Liver

Table S6. Sequences of primers used in this study

Primers for IVA cloning	n°	Sequence	
ATG3 to AAG F	p1461	CCAGCCCTGGCGAAGGCAACGGCGACGGG	
ATG3 to AAG R	p1462	CGCCAGGGCTGGGCAG	
ATG2 to AAG F	p1463	GCGGCCTCTGGGGCAAGGAGCACGCTGCC	
ATG2 to AAG R	p1464	TGCCCCAGAGGCCGC	
ATG1 to AAG F	p1465	GCCGCCCTATAAGTTGCCCGCCGCG	
ATG1 to AAG R	p1525	T ATA GGG CGG CGG CGC G	
ATG1 Perfect F	p1467	GCGCCGCCGCCCAAC<u>ATG</u>GTGCCCGCCGCGGC	
ATG1 Perfect R	p1468	GGGCGGCGGCGCGGGG	
primers for pcr from gDNA			
SRD5A1_F_60C	p1442	ctggGAGCTCCCTTTCTGCAGAGTCCCGGCAGTG	
SRD5A1_R1	p1446	ctggaccggtCAGGCGCTCCTCCGCCAC	
SRD5A1_R2	p1447	ctggaccggtgCAGGCGCTCCTCCGCCAC	
SRD5A1_R3	p1448	ctggaccggtagCAGGCGCTCCTCCGCCAC	
KM_SRD_Gb_Vec_FW		CATCCTACAAAGACTGCGAAAAGAAGCGC	Gibson assembly for longer section to include indels SRD5A1
KM_SRD_Gb_Vec_RV		AAAGGGAGCTCTGCTTATATAGACCTCCCAC	Gibson assembly for longer section to include indels
KM_SRD_Gb_Ins_FW		AAGCAGAGCTCCCTTTCTGCAGAGTCCCGGC	Gibson assembly for longer section to include indels
KM_SRD_Gb_Ins_RV		CGCAGTCTTTGTAGGATGCAGTTGGGCGC	Gibson assembly for longer section to include indels
KM_SRD_IVA_P2_FW		ATCCTCCACAAAGACTGCGAAAAGAAGCGC	Add 2 nucleotide
KM_SRD_IVA_P2_RV		CGCAGTCTTTGTGGAGGATGCAGTTGGGCGC	
