# Supplementary material for: Human SRD5A1 as a case of gene expression indel-resistance in triple-coding region
Source: Genome Biol. 2026 May 18;27:219. doi: 10.1186/s13059-026-04106-x (PMC13352861; doi:10.1186/s13059-026-04106-x)
Supplement: Supplementary file 7 — Additional file 7. Raw images of the gels used to design the figures. [file 13059_2026_4106_MOESM7_ESM.pdf]

Raw gel images

Figure 2, panel b

Biological replicate 1

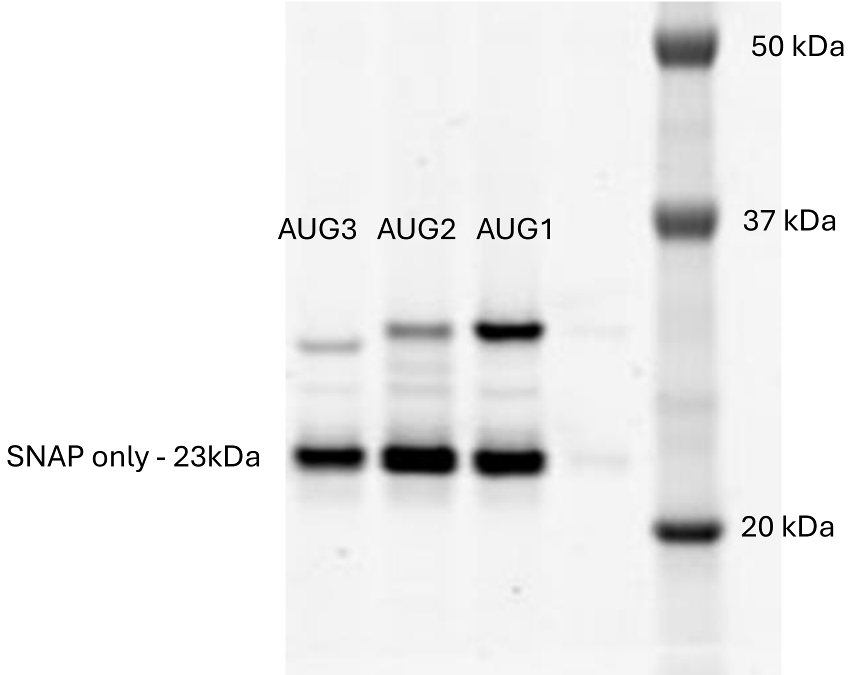

Figure 2, panel b

Biological replicate 2

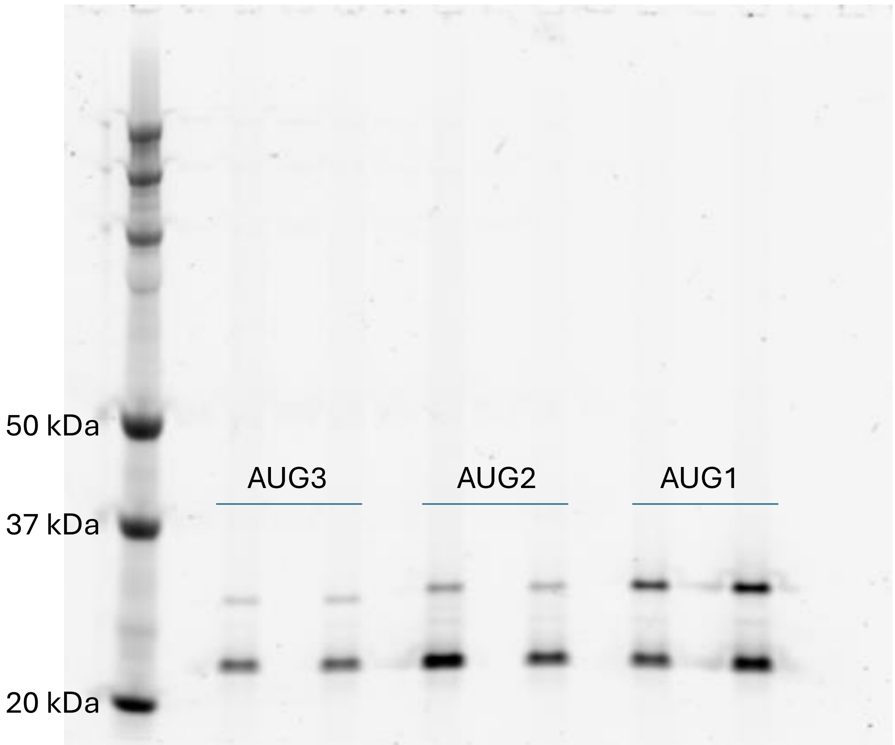

Figure 3, panel c

Biological Replicate 1

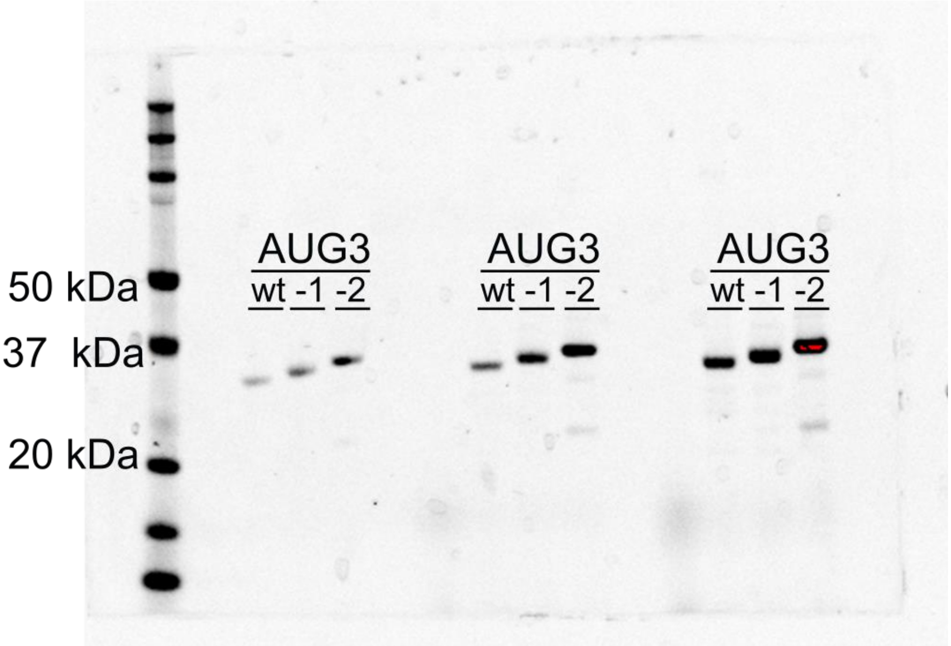

Figure 3, panel c

Biological Replicate 2

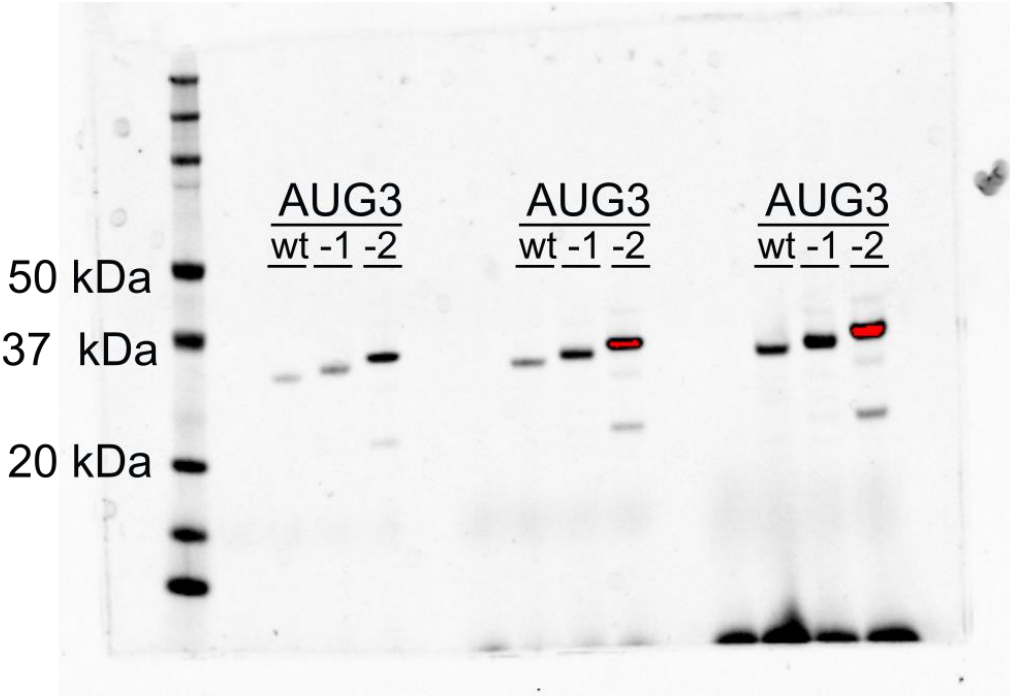

Figure S3

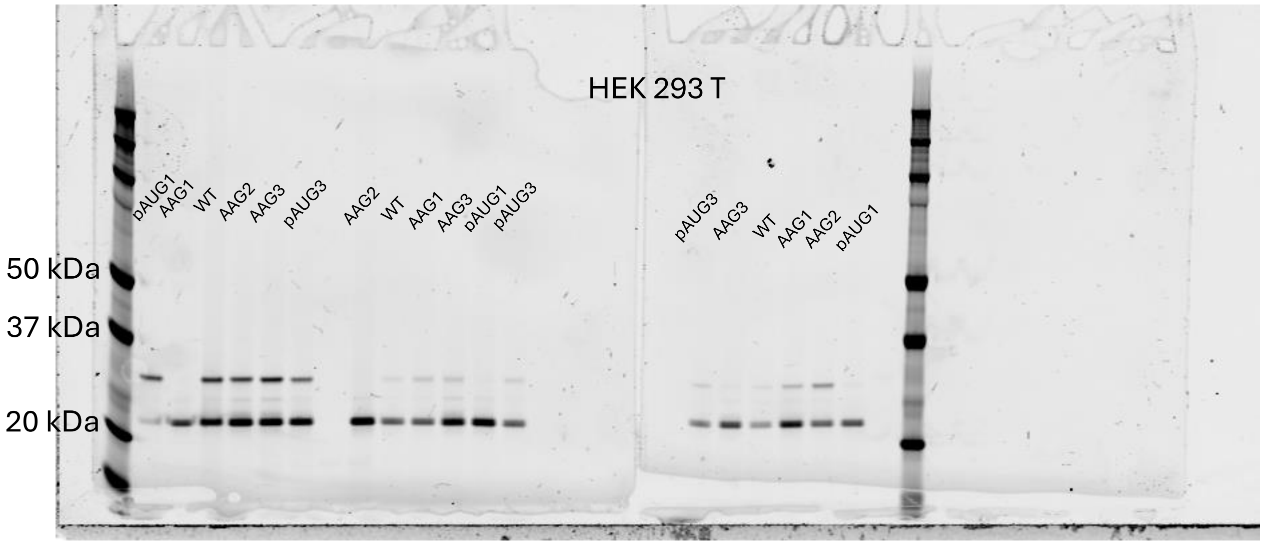
HEK 293 T
50 kDa
37 kDa
20 kDa
pAUG1
AAG1
WT
AAG2
AAG3
pAUG3
AAG2
WT
AAG1
AAG3
pAUG1
pAUG3
pAUG3
AAG3
WT
AAG1
AAG2
pAUG1
